# Long-term follow-up of intradermal injection of methylene blue for intractable, idiopathic pruritus ani

**DOI:** 10.1007/s10151-019-01934-x

**Published:** 2019-02-07

**Authors:** J. H. Kim, D. H. Kim, Y. P. Lee

**Affiliations:** 1 Department of Surgery, Ajou University School of Medicine, 164 Worldcup-ro, Youngtong-gu, Suwon, Gyeonggi-do 16499 Republic of Korea; 2 Department of Health Services Administration, University of Alabama at Birmingham, Birmingham, AL USA; 3 Department of Surgery, Hanvit Hospital, Suwon, Republic of Korea

**Keywords:** Intradermal injection, Methylene blue, Idiopathic pruritus ani

## Abstract

**Background:**

While various medical treatments such as topical steroid ointment, antihistamine agent, and sedatives have been used for treating idiopathic intractable pruritus ani, they are not long-term solutions, due to the high recurrence rate. The aim of this study was to determine the effect of methylene-blue intradermal-injection therapy for treating patients with idiopathic intractable pruritus ani. Symptom improvement and recurrence rates were determined with a long-term follow-up.

**Methods:**

A retrospective study was conducted from January 2011 to October 2013 on consecutive patients with intractable pruritus ani treated with methylene-blue intradermal injection. The therapy included 5 ml of 1% methylene blue and 15 ml of 1% lidocaine. Follow-up included a physical exam and satisfaction-score survey (1 = much worse, 2 = worse, 3 = no improvement, 4 = much better, 5 = gone completely) before treatment, 6 weeks after treatment, and 3 years after treatment to check patient status and recurrence rate.

**Results:**

Of 103 treated patients, 96 were able to attend the 6-week follow-up visit. There were 58 (60.4%) males and 38 (39.6%) females with a mean age of 48.34 ± 10.21 years. Their mean satisfaction score at 6 weeks was 4.23 ± 0.86. Of the total of 96 patients, 9 (9.4%) patients scored 3 or less in their satisfactions score at 6 weeks. 62 (64.6%) patients were evaluated 3-year post-treatment. The satisfaction score at 3 years after treatment was 4.74 ± 0.57. Besides the 9 patients who initially failed treatment, 4 of the remaining 53 patients scored 3 or less in their satisfaction score surveys. Thus, the recurrence rate at 3 years was 7.5% (4/53).

**Conclusions:**

Methylene-blue intradermal injection can result in a high symptom improvement rate with low recurrence rate for patients with idiopathic pruritus ani.

## Introduction

Pruritus ani is one of the most common symptoms in patients suffering from anal disease. Associated symptoms include skin breakdown, weeping, maceration, lichenification, and superinfection [1]. Furthermore, it can lead to mental distress. The mechanism of pruritus ani can be described as “itch–scratch–itch behavior”. That is, when the sensory nerve near the anus is stimulated, patient’s skin is most likely to become damaged due to the overwhelming need to excessively scratch the skin in the proximity of the anus [2].

Pruritus ani can be divided into two types: secondary to another condition, and idiopathic (IPA), which cannot be attributed to a specific cause [3]. IPA accounts for more than 50% of patients with this condition. Patients should avoid excessive soap usage, excessive paper towel usage, and excessive caffeine consumption. While various medical treatments such as topical steroid ointment, antihistamine agents, sedatives, and other local anesthetic therapies can be used, they are not long-term solutions and recurrence of symptoms is high. The aim of this study was to investigate the effect of methylene blue intradermal injection therapy on patients with intractable IPA. Symptom improvement and recurrence rates were determined with a long-term follow-up.

## Materials and methods

### Patients

**Fig. 1:**
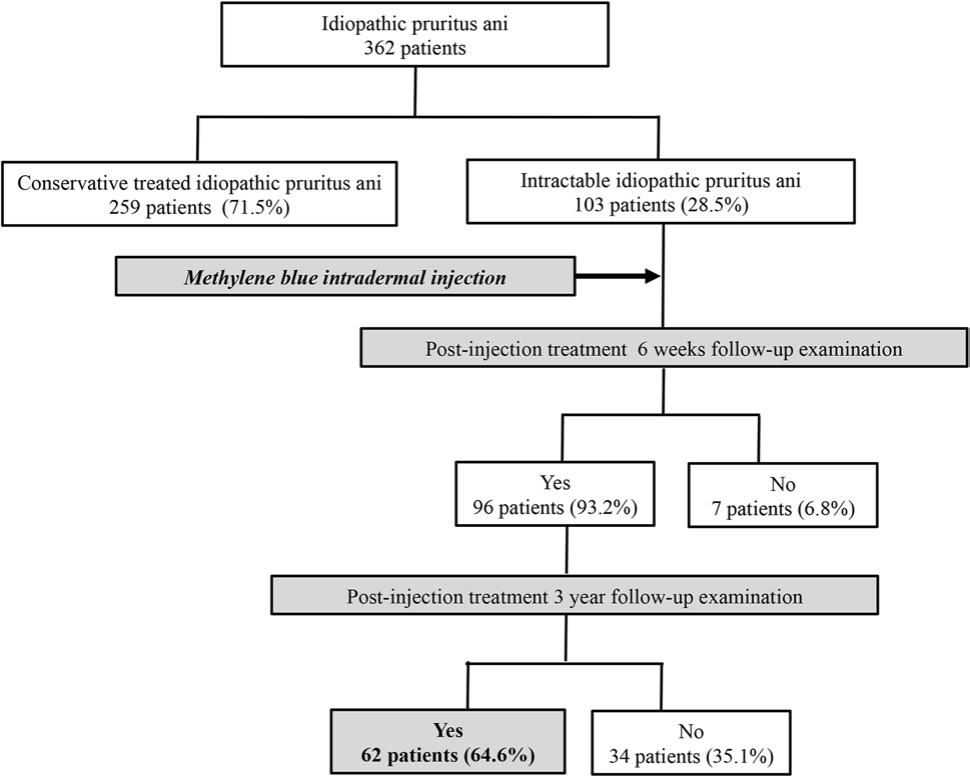
Flow diagram showing the follow-up of methylene blue intradermal injection therapy

A total of 362 patients who were treated  for anal itching at Hanvit Hospital, Republic of Korea, from January 2011 to October 2013 were considered for this study. They were investigated with anal ultrasonography, anorectal manometry, and anoscope to rule out causes of secondary pruritus ani including hemorrhoids, anal fissure, and fecal incontinence. Of these 362 patients, 259 were excluded from this study, because they got relief from conservative treatments such as sitz baths, diet control, local steroid ointment, and antihistamine agent. Patients who failed conservative treatment completed a symptom-score survey for which a visual analog scale (0 = no symptoms, 1–3 = mild, 4–7 = moderate, and 8–10 = severe) was used. A total of 103 patients recorded scores of 8–10 (severe group). These patients were selected to receive methylene-blue intradermal injection therapy. Ninety-six patients attended follow-up examination at 6 weeks after treatment. Of these 96 patients, 62 attended follow up at 3 years (Fig. 1). These cases were retrospectively reviewed using a prospectively designed database system.

### Technique and outcome measurements

**Fig. 2:**
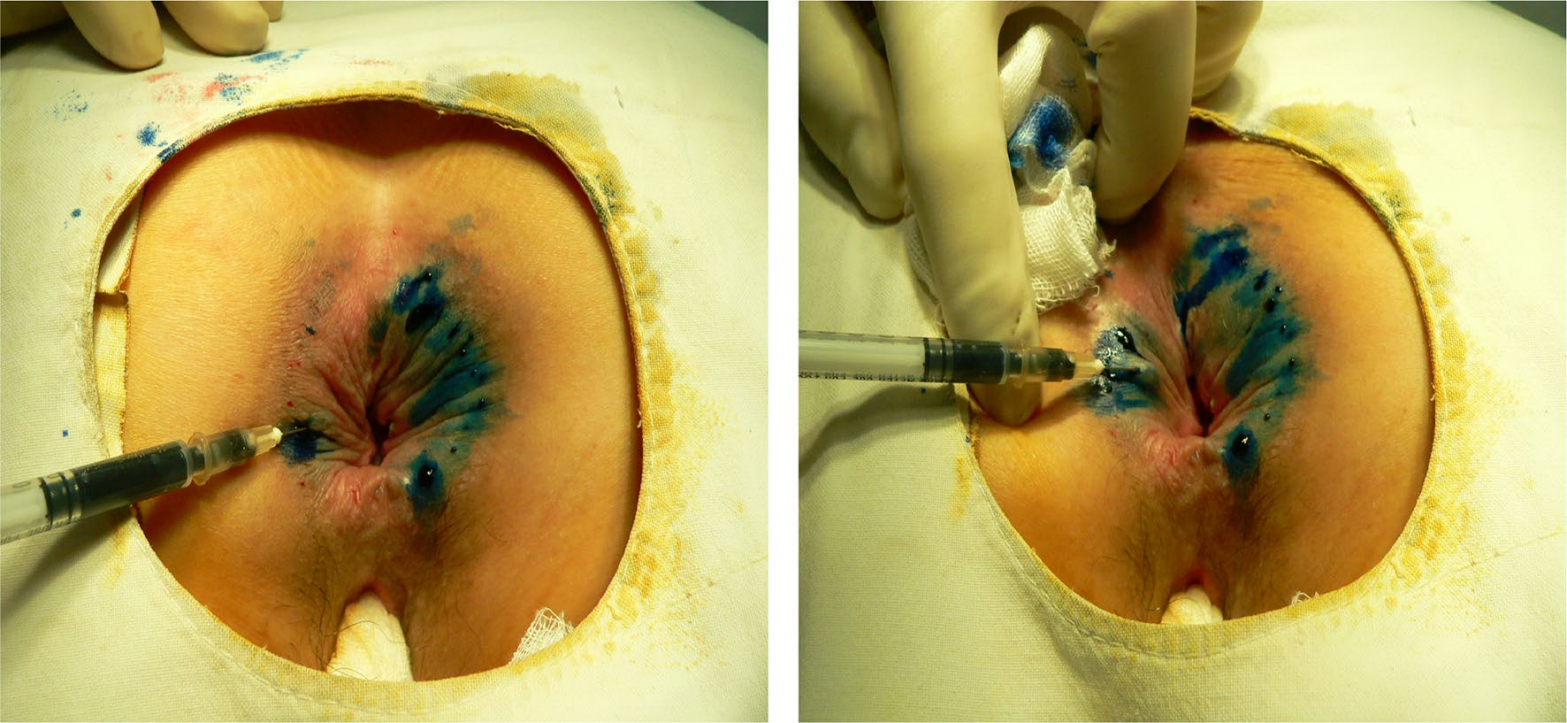
Technique of intradermal injection of methylene blue. Patient was under saddle block anesthesia in jack-knife position during treatment. Methylene blue intradermal injection therapy included 5 ml of 1% methylene blue and 15 ml of 1% lidocaine. A total of 20 ml was then injected into the perianal skin using a 22-gauge needle

Patients were placed in the prone jack-knife position during and given saddle block anaesthesia. The methylene-blue intradermal-injection therapy consisted of 5 ml of 1% methylene blue (Tera Pharmaceuticals, North Wales, PA, USA) and 15 ml of 1% lidocaine. Thus, a total of 20 ml was injected into the perianal skin of each patient (Fig. 2). A 22 gauge needle became our preference because of the difficulty in performing intradermal injection with larger needles. Anal physical examination and satisfaction-score survey [4, 5] (1 = much worse, 2 = worse, 3 = no improvement, 4 = much better, and 5 = gone completely) were used before treatment, at 6-week post-treatment, and at 3 years after treatment to check patient status and recurrence rate. If patients scored 3 or less the treatment was considered to have no effect [4, 5].

### Statistical analyses

IBM^®^ Statistics SPSS^®^ for Windows 23.0 (SPSS Inc., Chicago, IL, USA) was used for all data analyses. Data are expressed as mean ± standard deviation. An independent-samples *t* test was used for analysis. *A p* value < 0.05 was considered statistically significant.

### Ethics

This study was approved by the Institutional Review Board (IRB) of Korea National Institute for Bioethics Policy, Republic of Korea (No. P01-201808-21-009). The requirement for informed consent was exempted by the IRB due to its retrospective nature based on de-identified data.

## Results

Ninety six patients attended 6-week follow-up. There were 58 (60.4%) males and 38 (39.6%) females with a mean age of 48.34 ± 10.21 years. Their mean satisfaction score at 6-week post-treatment was 4.23 ± 0.86.

Of these 96 patients, 62 (64.6%) of them were evaluated at 3 years, 47 (75.8%) were males and 15 (24.2%) were females with a mean age of 45.56 ± 9.44 years. The mean length of time that these patients had suffered from pruritus ani was 9.26 ± 2.88 years (Table 1).

**Table 1:** Baseline characteristics of patients

Parameter	Value
No. of patients	62
Sex ratio, male:female	47:15
Age (years)	45.56 ± 9.44
Symptom duration (months)	9.26 ± 2.88

Data are expressed as numbers or absolute mean ± standard deviation

**Fig. 3:**
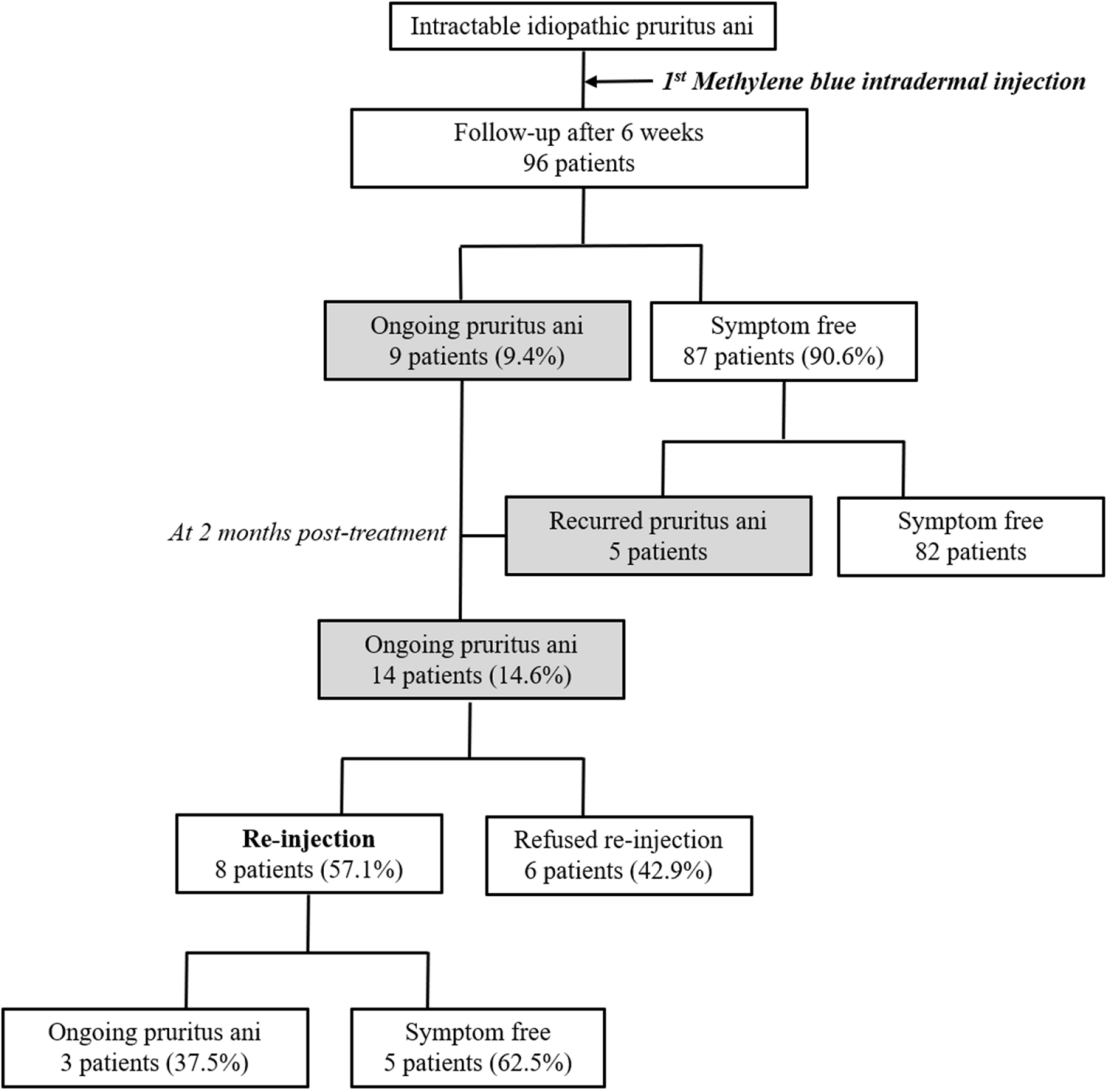
Follow-up results after methylene blue injection for idiopathic pruritus ani. The overall symptom improvement rate after methylene blue injection was 85.5% (53/62)

All patients scored 8 or above in the symptom score, with a mean score of 8.16 ± 0.45 (severe group). Fourteen patients (14.6%) had pain due to pruritus ani at 2-month post-treatment. Of these 14 patients, 8 (57.1%) had repeat treatment. Of these 8 patients, 5 showed symptomatic relief, while the remaining 3 patients continued to have pruritus ani. Of a total of 96 patients, 9 scored 3 or less in their satisfaction score surveys at 6-week post-treatment. Thus, the symptom improvement rate was 90.6% (Fig. 3). The satisfaction score 3 years after treatment was 4.74 ± 0.57. Nine patients who scored less than 3 in their satisfaction score surveys at 6 weeks after treatment, also participated in the post-treatment assessment at 3 years. Besides these 9 patients, only 4 of the remaining 53 patients scored 3 or less in their satisfaction score surveys. Thus, the recurrence rate was 7.5% (Table 2).

**Table 2:** Preoperative symptom score and postoperative satisfaction score

Parameter	Value	Recurrence
Preoperative symptom score^a^ (VAS)	8.16 ± 0.45	
Postoperative satisfaction score^b^ after 6 weeks	4.23 ± 0.86	9/62 (14.5)
Postoperative satisfaction score^b^ after 3 years	4.74 ± 0.57	4/53 (7.5)

Data are expressed as absolute mean ± standard deviation or numbers (percentage)

*VAS* Visual Analog Scale

^a^Symptom score: 0 = no symptom, 1–3 = mild, 4–7 = moderate, 8–10 = severe

^b^Satisfaction score: 1 = much worse, 2 = worse, 3 = no improvement, 4 = much better, 5 = gone completely

**Fig. 4:**
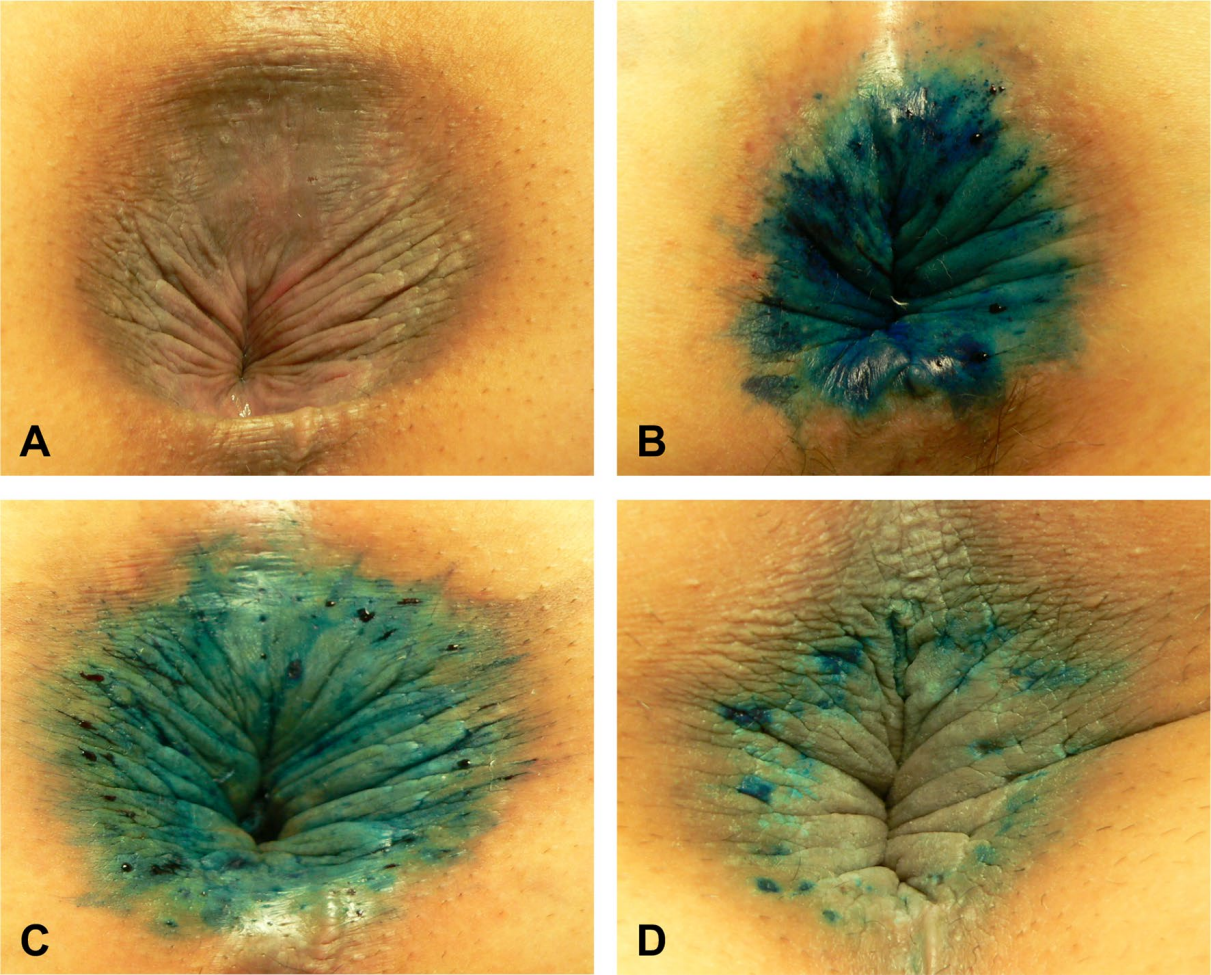
Progression of methylene blue injection. **a** Typical skin breakdown, maceration, and lichenification. **b** Immediately after methylene blue injection. **c** Two-week post-treatment. Tattooing started to disappear after 1–2 weeks. **d** Four-week post-treatment. After about 4 weeks, only small pin-point areas of tattooing were left. Blue color tattooing remained in the perianal skin (**c, d**)

Out of 96 patients that were available for the post-injection treatment follow-up examination at 6 weeks, 84 patients (87.5%) had signs of tattooing lasting up to 6 weeks during the analysis of tattooing durability and recurrence (Fig. 4). Out of 12 patients in whom signs of tattooing disappeared within 6 weeks, 8 scored less than 3 in their symptom scores (*p* < 0.001). These were 8 (88.9%) of the 9 patients that scored less than 3 in their satisfaction score surveys taken at 6 weeks after treatment.

Subcutaneous hematoma was found in 6 (9.7%) of the 62 patients. However, it spontaneously resolved after 2 weeks. There were no complications such as involuntary fecal seepage, perianal cellulitis, skin necrosis, or abscess formation after the treatment.

## Discussion

Pruritus ani is characterized by severe itching of the skin around the anus. Unfortunately, many of its causes are unknown. Pruritus ani is divided into the following two types: secondary pruritus ani due to a specific cause and IPA, which occurs without any specific cause [3]. In terms of secondary pruritus ani, variety of factors such as diabetes mellitus, obstructive jaundice, psoriasis, lichen planus, hemorrhoids, anal fissure, bacteria virus, fungus, parasite infection or reaction to coffee, chocolate, and beer can be the causes [1, 6–8]. Secondary pruritus ani, having specific causes, can easily be treated by physicians.

While some studies have reported that IPA constitutes approximately 25% of all pruritus ani cases [6], others have reported that 50% of such cases could be due to IPA [9]. Although excessive relaxation of the internal sphincter on anal manometry in response to rectal distension [10, 11] has been reported, the clinical relevance of these findings is uncertain. General treatments for IPA include dietary control, regular bowel habit, and elimination of anal irritants [12–14]. Drugs such as barrier ointments and antihistamine agents are also used. However, reports show that patients sometimes return to physicians due to recurring symptoms after treatment [14, 15].

Methylene blue intradermal injection therapy was first introduced as a treatment of IPA by Ryzhikh [16] in 1969. The methylene blue works by severing the nerve endings of the perianal skin’s unmyelinated C-fibers [17], which results in a decrease of the urge to scratch [2]. Eusebio et al. [18] reported on 23 patients being injected with 30 ml of 0.5% methylene blue with complete or partial success in 21 patients (91%). Of the 18 patients who responded and had a follow-up longer than 12 weeks (up to 9 years), 4 patients (22%) showed signs of recurrence. Farouk and Lee [19] reported on six patients injected with 10 ml of 1% methylene blue, along with 7.5 ml of 0.5% marcaine and 5 ml of normal saline. In five cases, there was a substantial reduction in symptoms after treatment, with marked regression of associated skin changes and only occasional minor symptoms of pruritus. The median duration of follow-up was 3 years. Three patients received second injections of methylene blue because of recurring symptoms at 1, 3, and 5 years. All three patients had initial numbness after the first injection. Borterill and Sagar [20] performed a study with 25 patients. Median duration of follow-up was 11 months. The researchers found that 16 (64%) of these patients had symptomatic relief after the first injection. On the other hand, 22 patients (88%) had signs of symptomatic relief after the second injection. In another study by Mentes et al. [21] of 30 patients examined at 1 and 6 months, and then on a yearly basis. 24 (80%) had symptom relief after the first injection, while 28 patients (93.3%) had symptom relief after the second injection. At 6 months, 3 recurrences were noted, with an overall success rate of 83.3% (25/30). At 1 year, 23 patients (76.6%) were free of symptoms. Three of the five cases of recurrence noted at the 1-year follow-up developed in the group of five patients who went through a second methylene blue injection. Although only five patients completed 2-year follow-up, no additional recurrences were been noted. Sutherland et al. [4] performed a study on 49 patients with follow-up at 4 and 8 weeks after treatment. They found that 96% of their patients had symptom relief, while in 57% were IPA completely resolved after the first injection. Furthermore, all four patients who received the second injection became symptom free. The symptoms of seven patients who had continence changes resolved completely within 10 days to 6 weeks. In contrast, Samalavicius et al. [5] reported on 10 patients with IPA who had resolution of symptoms after being injected with 1% methylene blue solution and were followed up for a median of 47 months. Anal itching recurred in eight patients. Four out of eight patients stated that anal itching was less severe when it reoccurred. All of the eight patients with recurrence needed some kind of additional conservative treatment. Methylene blue injection was not repeated in any of these patients; however, 6 of the 10 patients considered their symptoms resolved.

The present study revealed that 48 (77.4%) of 62 patients had symptom relief after the first injection, while 53 (85.5%) patients had symptom relief after the second injection. These results are similar to the ones listed above.

We found a lower recurrence rate with prolonged methylene blue tattooing around the anus. Our study showed that 48 patients with tattooing lasting 4–6 weeks had symptom relief without recurring symptoms. Alternatively, in those patients, whose tattooing disappeared in less than 2 weeks, the recurrence rate was high. Other studies have also reported that the duration of tattooing for 4–6 weeks correlated with low recurrence rates [20, 21]. We believe that the duration of tattooing depends on the width of the needle used for intradermal injection. Although 5 ml of 1% methylene blue and 15 ml of 1% lidocaine were injected in all cases, we believe that in some occasions, the injection was not intradermal but subcutaneous, leading to more rapid disappearance of tattooing. At the beginning of our experience, we used various types of needles with different gauges. However, we observed that with needles thicker than 22 gauge, it was not possible to make precise intradermal injections and this resulted in, deep subcutaneous injections, making the tattooing to disappear much more quickly. On the other hand, using needles thinner than 22 gauge made it difficult for sufficient amount of medication to be injected.

Some studies have shown that mixing a local anesthetic agent such as lidocaine with methylene blue can decrease the pain level of patients during or after treatment [18]. However, in our experience, a regional block is necessary in addition to the local anesthetic mixed with methylene blue. At first, three patients underwent the treatment without regional block. However, these patients felt pain either during or after the treatment. Thus, saddle-block anesthesia was implemented. The reason for physicians to inject lidocaine after application of saddle-block anesthesia is mainly to decrease the pain level after the treatment.

Previous studies have shown that complications such as perianal cellulitis, skin necrosis, and abscess formation can occur after treatment [18, 19]. Temporary fecal seepage was also observed in 4% of patients within 2 days after treatment [20]. However, none of these complications was evident in the present study. Although six patients had post-treatment subcutaneous hematoma, the hematomas resolved spontaneously within 2 weeks after treatment.

One major limitation of the present study is that out of 103 patients who had methylene blue intradermal injection, only 62 patients (60.2%) were available for the 3-year follow-up.

## Conclusions

In IPA patients, intradermal injection of methylene blue can lead to a high rate of symptom improvement and a low recurrence rate. Since repeat treatment is possible and the probability of complications is very low, methylene-blue intradermal injection can be considered as an appropriate treatment method for IPA patients.

## Data Availability

The original anonymous data set is available upon request to the corresponding author at conan1602@aumc.ac.kr
